# Direct Stimulation of Human Hippocampus During Verbal Associative Encoding Enhances Subsequent Memory Recollection

**DOI:** 10.3389/fnhum.2019.00023

**Published:** 2019-02-05

**Authors:** Soyeon Jun, June Sic Kim, Chun Kee Chung

**Affiliations:** ^1^Department of Brain and Cognitive Sciences, Seoul National University, Seoul, South Korea; ^2^Research Institute of Basic Sciences, Seoul National University, Seoul, South Korea; ^3^Department of Neurosurgery, Seoul National University Hospital, Seoul, South Korea

**Keywords:** brain stimulation, memory enhancement, recollection, hippocampus, theta power, lateral temporal cortex

## Abstract

Previous studies have reported conflicting results regarding the effect of direct electrical stimulation of the human hippocampus on memory performance. A major function of the hippocampus is to form associations between individual elements of experience. However, the effect of direct hippocampal stimulation on associative memory remains largely inconclusive, with most evidence coming from studies employing non-invasive stimulation. Here, we therefore tested the hypothesis that direct electrical stimulation of the hippocampus specifically enhances hippocampal-dependent associative memory. To test this hypothesis, we recruited surgical patients with implanted subdural electrodes to perform a word pair memory task during which the hippocampus was stimulated. Our results indicate that stimulation of the hippocampus during encoding helped to build strong associative memories and enhanced recollection in subsequent trials. Moreover, stimulation significantly increased theta power in the lateral middle temporal cortex during successful memory encoding. Overall, our findings indicate that hippocampal stimulation positively impacts performance during a word pair memory task, suggesting that successful memory encoding involves the temporal cortex, which may act together with the hippocampus.

## Introduction

The hippocampus plays a pivotal role in associative memory (Olsen et al., 2012; Yonelinas, 2013), serving as a hub that supports the binding of information (Battaglia et al., 2011); it is thus regarded as a core region for stimulation in attempts to manipulate the memory circuit (Eichenbaum et al., 2007). Some previous studies have reported that direct hippocampal stimulation exerts negative effects (Coleshill et al., 2004; Lacruz et al., 2010; Jacobs et al., 2016; Goyal et al., 2018) or no effect (Suthana et al., 2012) on memory, whereas others have reported positive effects (Berger et al., 2011; Hampson et al., 2012; Fell et al., 2013). However, several non-invasive stimulation studies have reported that hippocampal stimulation enhances paired associative memory (Wang et al., 2014; Wang and Voss, 2015). In one recent study, hippocampal-targeted TMS enhanced associative memory. In contrast, item memory was unaffected, demonstrating a selective influence on associative memory vs. item memory (Tambini et al., 2018). Specifically, multiple-day electromagnetic stimulation has been shown to enhance memory recollection, by exerting network-level effects on memory precision (Nilakantan et al., 2017). However, as current evidence mostly comes from indirect stimulation of the hippocampus, the effect of direct hippocampal stimulation on associative memory has yet to be determined.

Recollection is defined as the retrieval of contextual details associated with a previously experienced event (Yonelinas, 2013). The cognitive process of recollection involves a set of brain regions termed the “recollection network” (Rugg and Vilberg, 2013). Numerous studies have shown both critical and necessary roles for structures outside of the medial temporal lobe (MTL) in memory (Buzsáki, 1996; Eichenbaum, 2000; Poldrack et al., 2001; Ritchey et al., 2015; Moscovitch et al., 2016). In particular, the lateral temporal cortex plays a key role in episodic memory processing (Chao et al., 1999). A recent study using lateral temporal cortical stimulation demonstrated enhanced verbal memory performance, presenting it as a viable target for exploring memory enhancement (Kucewicz et al., 2018). Furthermore, functional imaging changes have been observed in this region during the encoding stage of explicit verbal memory (Casasanto et al., 2002; Fletcher and Tyler, 2002). Indeed, neuronal activity in the human lateral temporal cortex has been shown to increase during the learning of associations between word pairs, suggesting that human associative learning is related to the activity of a specific population of “association” neurons (Ojemann and Schoenfield-McNeill, 1998; Ojemann et al., 2002, 2009).

Previous human intracranial electroencephalogram (iEEG) studies have reported that neural oscillatory changes in memory-related neocortical regions are accompanied by successful memory formation relative to unsuccessful encoding (Burke et al., 2013; Watrous et al., 2013; Lega et al., 2016). Specifically, low-frequency oscillations entrain the rhythm of behavioral tasks to optimize energy-efficient performance (Lakatos et al., 2008; Daitch et al., 2013), while changes in theta power enhance episodic memory (Rutishauser et al., 2010; Addante et al., 2011; Lega et al., 2012; Backus et al., 2016; Sweeney-Reed et al., 2016). In the present study, we sought to investigate changes in activity within the neocortical region (i.e., the lateral temporal cortex) during encoding. We presumed that low-frequency activity in the temporal neocortex reflects the effects of stimulation-induced activity, indicating that verbal episodic memory encoding involves a network of neocortical structures that may act interdependently with the hippocampus.

To test this hypothesis, we applied direct hippocampal stimulation in surgical patients with epilepsy based on methods described in previous studies (Suthana et al., 2012; Jacobs et al., 2016; Hansen et al., 2018). However, we utilized a stimulation current with a higher amplitude (2 mA) than that used in the aforementioned studies (0.1–1.5 mA). In addition, we adopted a word pair memory task that involved recruitment of the hippocampus during encoding (Axmacher et al., 2008; Hamani et al., 2008). To the best of our knowledge, the present study is the first of its kind utilizing a word pair memory task, which enabled us to compare the ability to remember a learning episode (recollection) with the capacity to judge items as familiar (familiarity; Manns et al., 2003; Mickes et al., 2010).

## Materials and Methods

### Patients

The present study included six patients (four women; mean age: 33.6 ± 10.8 years) with drug-resistant epilepsy who had been implanted with intracranial electrodes to determine the area of the seizure onset zone. Human subjects: this study was approved by the Institutional Review Board of Seoul National University Hospital (H-1407-115-596). All subjects provided written informed consent to participate in the study. A single mid-hippocampal electrode had been implanted in each patient based on the expertise of neuroradiologists experienced in neuroanatomy (Table 1).

**Table 1:** Patient demographics, clinical characteristics, electrode locations, and stimulation parameters.

Patient	Demographics		Clinical characteristics				Stimulation parameter	
	Age	IQ/MQ	Seizure onset	Pathology	Resection	Epilepsy type	Anode	Cathode
1	25–30	97/112	ATG, TP	PHG reactive gliosis	PHG	TLE	R. mHP	LWM
2	25–30	78/81	TP, STG	Temporal lobe FCD	L. ITG	TLE	L. mHP	LWM
3	20–25	110/60	Amygdala	FCD heterotopia	PHG, Amygdala	TLE	R. mHP	LWM
4	30–35	91/90	STG	HP neuronal loss	ATL, AH	TLE	L. mHP	LWM
5	50–55	77/94	PHG	DG dispersion, HP neuronal loss	HP	TLE	L. mHP	LWM
6	25–30	85/111	OFC	HP neuronal loss	Amygdala, PHG	TLE	R. mHP	LWM

*Abbreviations: IQ, intelligence quotient; MQ, memory quotient; R., right; L., left; HP, hippocampus; mHP, mid-hippocampus; LWM, limbic white matter; PHG, parahippocampal gyrus; DG, dentate gyrus; ATG, anterior temporal gyrus; STG, superior temporal gyrus; ITG, inferior temporal gyrus; TP, temporal pole; TLE, temporal lobe epilepsy; FCD, focal cortical dysplasia; ATL, anterior temporal lobe; AH, anterior hypothalamus; OFC, orbitofrontal cortex. Patient demographic data are presented together with clinical observations regarding identified seizure onset zones, pathology in patients who underwent corresponding surgery, and neuropsychological test results. A clinical psychologist employed the Wechsler Adult Intelligence Scale-Korean version (K-WAIS-IV) and the MQ of the Rey-Kim Memory test to assess each patient’s IQ. Anode and cathode locations indicate brain regions of stimulation in each patient. In all patients, the stimulation location was either the left or right mid-hippocampus. The mean current was 2 mA, and the mean charge density was 360 μC/cm^2^/phase*.

### Electrode Localization

**Figure 1:**
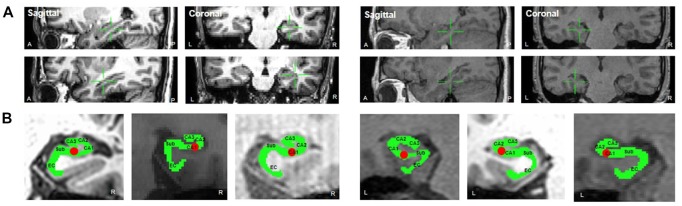
**(A)** Location of stimulation. The green crosshair denotes the location of the stimulation electrode in the right mid-hippocampus (sagittal and coronal, respectively) in Patient 1 (left two panels, anode and cathode, respectively) and in the left mid-hippocampus in Patient 2 (right two panels). **(B)** The hippocampal subregions in each patient and one hippocampal electrode in the region of interest.

Electrodes were implanted for clinical purposes only. Depending on clinical need, electrodes (AdTech Medical Instrument Corporation, Racine, WI, USA) were either placed at depth within the MTL (platinum, surface area of 0.059 cm^2^, placed 6 mm apart) or positioned for subdural electrocorticography (ECoG) on the cortical surface (diameter of 4 mm, placed 10 mm apart) with stainless steel contacts. Prior to electrode implantation, each patient underwent an magnetic resonance imaging (MRI) scan. Additional MRI and CT scans were performed following electrode implantation. Patients underwent preoperative MR imaging in a Magnetom Trio, Magnetim Verio 3-tesla (Siemens, München, Germany) or Signa 1.5-tesla scanner (GE, Boston, MA, USA). CT images were recorded using a Somatom sensation device (64 eco; Siemens München, Germany). Each patient had at least one hippocampal electrode in the region of interest. Target hippocampal electrodes for stimulation (anode) were inserted into the mid-body of the hippocampus gray matter using a temporo-lateral approach. Given the electrode contact space within the MTL, the pairing electrode (cathode) of stimulation was identified in the temporal white matter (Figure 1A). A neuroradiologist and neurosurgeon experienced in neuroanatomical localization identified bipolar pairs of electrodes within medial lobe sites, including the hippocampus, based on thin-section post-implantation CT scans and cross-sectional images. For visualization, individual preoperative MR images and postoperative CT images were co-registered as previously described (Avants et al., 2008), using CURRY software version 7.0 (Compumedics Neuroscan, Charlotte, NC, USA). In addition, the hippocampal subregions were localized using a manual segmentation process (Boccardi et al., 2015; Figure 1B).

### Stimulation Procedure

Stimulation was only administered during the encoding phase, as in previous studies (Suthana et al., 2012; Jacobs et al., 2016). During the task, the stimulation was configured to provide a bipolar stimulation between a pair of neighboring electrodes. Stimulation was delivered with a Grass S12X cortical stimulator (Natus, Warwick, RI, USA), using the following parameters: a frequency of 50 Hz, a balanced biphasic squared-wave pulse of 300 μs per phase, a 2-mA current, and total energy between 30 and 57 μC/cm^2^/phase. These parameters have been demonstrated to be safe and well-tolerated in patients with epilepsy (Kuncel and Grill, 2004; Boon et al., 2007), and the energy level was kept well below the safe maximum used for long-term and short-term stimulation (30 and 57, respectively; Agnew and McCreery, 1990; Gordon et al., 1990). The impedance of the depth electrode was always between 1 and 10 KΩ. Patients could not indicate when stimulation was applied.

### Verbal Associative Memory Task

**Figure 2:**
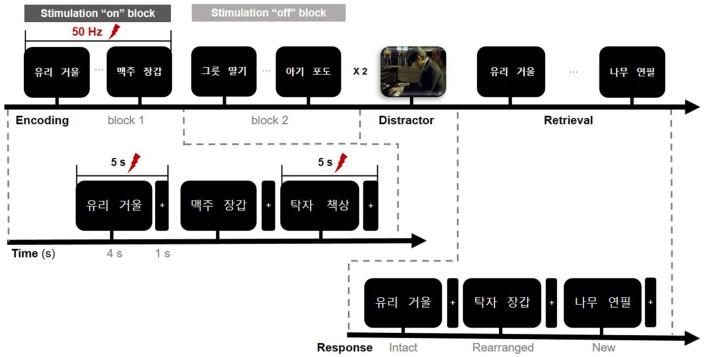
Timeline of the memory paradigm. The whole task consisted of three study periods: encoding, rest (distractor), and retrieval. Stimulation was delivered on-and-off at 50 Hz for 5 s during the encoding phase only and was randomly assigned to one of the two blocks in a single session. Lightning bolts denote periods of stimulation. In the encoding phase, the first word pair on the screen denotes “glass” (left) and “mirror” (right).

All stimuli were presented on a laptop computer, with Stim 2 Gentask (Neuroscan, Charlotte, NC, USA) used to present the word stimuli. We used a word pair memory task (Figure 2), which has been associated with recruitment of the MTL during memory encoding (Axmacher et al., 2008; Hamani et al., 2008). All word pairs consisted of two concrete Korean nouns with a mean frequency value of 105.11 (SD = 3.35, IQR = 122.5) according to the Korean Category Norms: Survey on Exemplar Frequency Norm, Typicality, and Features (Rhee, 1991) and the 2nd version of the Modern Korean Words database (Kim, 2005). Prior to the experimental session, a brief practice block of trials was administered to ensure that patients understood the task. For encoding, patients were asked to study 120 pairs over two sessions. Each word-pair trial began with a fixation cross appearing on the screen for 1 s, followed by the pair that was displayed for 4 s. Each session consisted of two blocks. The stimulation was randomized to one of the two blocks in each session. To ensure deep encoding, patients were instructed to report, by pressing a keyboard button with their index finger, whether they judged the word on the screen as “pleasant” or “unpleasant” (de Vanssay-Maigne et al., 2011). Following the encoding period, patients rested for 10 min and watched a 4-min video for distraction, to prevent inner rehearsal. During the retrieval period, patients were presented with 160 word pairs, including 40 new word pairs, 60 intact word pairs, and 60 rearranged word pairs. No word appeared twice, and patients were not exposed to the same experimental task more than once. Patients were asked to respond, by pressing one of three keyboard buttons, as accurately and quickly as possible depending on the presentation of the pairing options: same pairing (“intact”), altered word pairing (“rearranged”), or new pairing (“new”).

### Data Acquisition and Analysis

We recorded iEEG data including depth and ECoG using a 64-channel digital video monitoring system (Telefactor Beehive Horizon with an AURA LTM 64- and 128-channel amplifier system; Natus Neurology, West Warwick, RI, USA) digitized at a sampling rate of 1,600 Hz and filtered from 0.1 to 60 Hz. These iEEG data were analyzed using MATLAB software (version 2015b, Mathworks, Natick, MA, USA). We first performed manual artifact rejection of the signal for every electrode. Channels affected by artifacts were excluded from subsequent analyses. For example, individual stimulus response trials were precluded if there were any motion artifacts. Signals exhibiting stimulation artifacts and epileptic-form spikes were also excluded from further analyses. The recorded data were re-referenced to the common average reference (CAR). To quantify specific changes in theta rhythm during stimulation for the encoding period of the memory task, we applied time-frequency analysis and used Morlet wavelet transformation (wave number: 2.48) to obtain a continuous-time complex value representation of the signal. Transformed data were squared to calculate power and normalized by the mean and standard deviation of the pre-stimulus baseline power (i.e., resting periods prior to the task) of each frequency. Data were then epoched with a window of 0–4 s from the onset of paired-word trials and aligned with 50-Hz stimulation, beginning at the onset of the memory task. In our experiment, stimulation “on” and “off” blocks were conducted separately. To avoid direct stimulation artifacts, the OFF periods of trials in the stimulation “on” block were used for the stimulation trials (Figure 2). Stimulation trials thus included 30 stimulation trials from the total 60 word pairs across the two stimulation “on” blocks, while the non-stimulation trials included 60 word pairs across the two stimulation “off” blocks. We compared the averaged power of each condition across a frequency range of 3–7 Hz for correctly and incorrectly encoded memory items. For visualization, normalized data were averaged across all trials for correct and incorrect trials, according to each condition. We extracted *t*-scores to perform independent two-sample *t*-tests.

### Statistical Analysis

Statistical tests were performed using the Statistical Package for Social Sciences v12.0 K (SPSS) and MATLAB (Mathworks). Our primary measurement of memory performance was the percentage of correctly recognized trials in each block. Paired non-parametric rank-sum *t*-tests were used to compare behavioral performance between conditions. The level of statistical significance was set at *p* < 0.05. For activity in the lateral temporal cortex, independent two-sample t-statistics (***p < 0.01 or **p < 0.05) were used to compare the average power amplitudes of iEEG waveforms between correctly and incorrectly recognized trials during the stimulation “on” and “off” periods. Prior to significance testing, normality was assessed using the Lilliefors test (*p* > 0.01, for all datasets). For multiple comparisons among theta power levels, the Bonferroni correction procedure was employed.

## Results

### Enhancement of Memory Recollection

**Figure 3:**
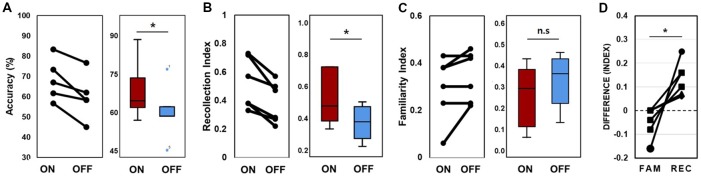
Memory performance. **(A)** Lines connect each patient’s “on” and “off” data point (left); data from all six patients were averaged (right). Accuracy refers to the proportion of correctly recognized words during the stimulation “on” and stimulation “off” periods. **(B)** Lines connect each patient’s “on” and “off” data for the recollection index (left); data were averaged across all six patients (right). **(C)** Familiarity index **(D)**. Difference scores of the stimulation effect of the two conditions. FAM indicates the familiarity index and REC indicates the recollection index. *p < 0.05. Error bars indicate the standard error of the mean (SEM). n.s., not significant.

In the behavioral analysis, we quantified the effect of stimulation on memory performance in two ways: first, we determined the hit rate of associative memory; second, we examined whether stimulation affected the ability to remember a learning episode (recollection) or the capacity to judge items as familiar (familiarity). At the behavioral level, individual memory performance was measured during the encoding phase of the word pair memory task during “on” and “off” hippocampal stimulation. The proportion of intact word pairs correctly identified as intact and rearranged word pairs correctly identified as rearranged were regarded as correct responses. The average accuracy values for all patients suggested that stimulation induced overall improvements in pair recognition memory (Figure 3A, Wilcoxon rank sum test, *p* = 0.027). Consistent increases in total raw scores and word accuracy were observed during the “on” period. The stimulation order was randomized across the six patients and we could not find any behavioral tendency regarding stimulation order (Table 2).

**Table 2:** Word pair memory task behavioral results.

Subject	Stimulation side	Stimulation block	Proportion of correct pairs (ON/OFF)		Proportion of correct pairs	Performance (%)	
			“intact”	“rearranged”	“new”	ON	OFF
Subject #1	Right	1,3	1.04	1.09	0.6	62	58
Subject #2	Left	1,4	1	2.17	0.63	57	45
Subject #3	Right	2,3	1.04	1.14	0.78	67	62
Subject #4	Left	2,4	1.04	1.14	0.9	83	77
Subject #5	Left	1,4	1.05	1.07	0.53	62	58
Subject #6	Right	2,3	1.06	1.47	0.83	73	58
Averaged		**-**	**1.04**	**1.35**	**0.71**	**67**	**60**

*Word pair memory task scores were obtained with stimulation randomly assigned to the “on” of “off” condition. Stimulation side indicates brain regions where each subject received stimulation. The proportion of correct pairs of stimulation is shown across “on” and “off” stimulation conditions for intact and rearranged pairs. The proportion of correctly identified new pairs. Performance indicates the proportion of correctly recognized pairs (intact pairs correctly identified as intact and rearranged pairs correctly identified as rearranged) during “on” and “off” stimulation. Average across all six patients is shown in bold*.

As word-to-word associations may reflect one of two independent types of retrieval (recollection of a specific experience or a sense of familiarity; Giovanello et al., 2006; Quamme et al., 2007), we used a similar procedure to measure hippocampal-dependent memory to obtain pure estimates of general memory and familiarity (Yonelinas and Jacoby, 1995; Cohn and Moscovitch, 2007; Hamani et al., 2008).

We calculated the recollection index, which reflects the hit rate of associative memory (i.e., intact pairs correctly identified as intact and rearranged pairs correctly identified as rearranged) minus the false alarm rate in associative memory (i.e., rearranged pairs identified as intact). The familiarity index was calculated by dividing the false alarm rate in associative memory (i.e., rearranged pair identified as intact) by 1 minus the recollection index. Comparisons between the recollection and familiarity indices revealed that only recollection was markedly and consistently increased in all patients during the stimulation “on” period (Figures 3B,C, Wilcoxon rank sum test, *p* = 0.027 and *p* = 0.068, respectively; Table 3). We observed significant differences in recollection but not familiarity indices; based on the stimulation, the difference index for the two conditions showed statistical significance (Wilcoxon rank sum test, *p* = 0.028, Figure 3D).

**Table 3:** Recollection vs. familiarity index for stimulation “on” and “off”.

Subject	Stimulation block	d’ (β)	Recollection^a^		Familiarity^b^	
		Stimulation “on”	Stimulation “off”	Stimulation “on”	Stimulation “off”
Subject #1	1,3	0.51 (1)	**0.33**	0.27	0.43	0.43
Subject #2	1,4	1.01 (1)	**0.38**	0.22	0.30	0.30
Subject #3	2,3	0.98 (1)	**0.57**	0.50	0.11	0.13
Subject #4	2,4	0.8 (1)	**0.75**	0.55	0.38	0.46
Subject #5	1,4	1.23 (1)	**0.38**	0.28	0.28	0.42
Subject #6	2,3	0.7 (1)	**0.72**	0.47	0.06	0.22
Averaged	-	0.87 (1)	**0.52**	0.39	0.30	0.34

*Abbreviation: d’ indicates the discriminability index, and β denotes the decision bias index. Associative verbal memory tasks were performed with stimulation randomly assigned to the “on” or “off” condition. Recollection^a^ indicates the proportion of correctly recognized intact and rearranged words—the proportion of falsely recognized words. Familiarity^b^ is the percentage of falsely recognized rearranged words in intact pair recognition/[1-recollection]. Increased indices during stimulation are shown in bold*.

### Stimulation Induces Theta Increases in the Lateral Middle Temporal Cortex During Correctly Remembered Encoding Trials

To assess whether neural population signals from the temporal neocortex regions change depend on the episodic memory changes, we performed time-frequency analyses for each stimulation condition. We tested whether the lateral middle temporal cortex displayed stimulation-induced theta activities; as indication of the role of theta activation in memory performance, we compared oscillatory power between correctly encoded trials and incorrectly encoded trials.

**Figure 4:**
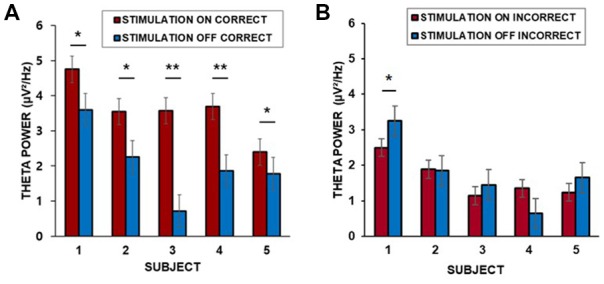
**(A)** Individual differences in theta power in the middle temporal cortex over 4 s for correctly remembered words during the “on” and “off” periods. **(B)** Individual differences in theta power in the middle temporal cortex over 4 s for incorrectly remembered words during the “on” and “off” periods. ***p < 0.01, **p < 0.05, corrected. Error bars indicate the standard error of the mean (SEM).

We found that the baseline corrected theta power (3–7 Hz) in the middle temporal cortex averaged across trials for each patient was greater during the “on” period than during the “off” period. Corrections for multiple comparisons revealed that the theta amplitude in the lateral temporal cortex significantly increased during the presentation of word stimuli (4 s) during the “on” period in all five patients for which data from lateral temporal recording electrodes were available (two-sample *t*-test, Bonferroni-corrected: *p* = 0.035; *p* = 0.002; *p* = 0.032; *p* = 0.008; *p* = 0.042, for patients 1–5, respectively, Figure 4A). Patient 6 did not have lateral temporal electrodes. We also compared incorrectly remembered encoding trials between “on” and “off” stimulation periods. There was no significant increase in theta power in the lateral temporal cortex during the stimulation “on” period (*p* = 0.040; *p* = 0.089; *p* = 0.075; *p* = 0.051; *p* = 0.067, for patients 1–5, respectively, Figure 4B).

**Figure 5:**
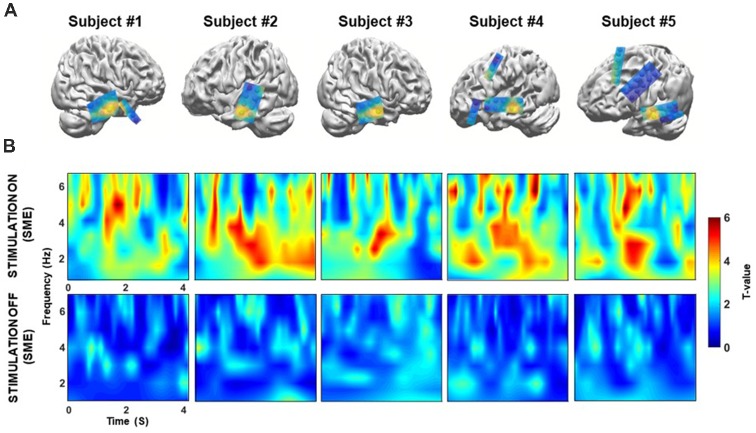
**(A)** Location of the target in the lateral temporal cortex in the sagittal plane following co-registration of preoperative high-resolution MRI and postoperative CT images (not illustrated), for patients 1–5, respectively. Spheres indicate the location of the recording site. Topographical maps of differences in theta power between the correct and incorrect responses during both the “on” and “off” condition. The yellow sphere denotes locations in which significant increases in theta power were observed in the “on” condition that were higher than those observed in the “off” condition. The yellow sphere indicates the region for the time-frequency map in panel B. Some electrode grids/strips were excluded because they were not visible. **(B)** Time-frequency map comparing correctly and incorrectly remembered pairs during the “on” and “off” period for five patients. On the x-axis, 0 s indicates the onset of the memory task. During the “on” condition, theta (3–7 Hz) power was significantly increased during the 4 s of word presentation (*p* < 0.05, corrected) for successfully recognized trials. In contrast, no significant increases in theta power were observed during the “off” condition (*p* > 0.05, corrected).

Next, we confirmed that these theta power differences could only be detected in the temporal cortex. We extracted the theta power from all electrodes during the memory encoding phase of the “on” and “off” stimulation periods, and then tested for differences (Figure 5A). Topographical maps were constructed to visualize differences in theta power between the correct and incorrect responses for both the “on” and “off” conditions for each patient’s electrodes. The results show that prominent theta power differences are only found in the middle temporal cortex. The individual normalized time-frequency topographical maps for the stimulation “on” period showed an overall increase in theta power in the middle temporal cortex (two-sample *t*-test, Bonferroni-corrected: *p* < 0.05, Figure 5B, upper panel). Conversely, no significant increase in theta power was observed for the contrast map in the middle temporal cortex during the stimulation “off” period (two-sample *t*-test, Bonferroni-corrected: *p* > 0.05, Figure 5B; bottom panel).

## Discussion

In the present study, we examined the effect of hippocampal stimulation on a word pair memory task. Our findings indicate that such stimulation enhanced memory performance in individual patients, and that this increase in pair recognition was reflected in recollection estimates rather than familiarity estimates. Furthermore, our findings show that theta power increased in the middle temporal cortex during the stimulation condition (i.e., during encoding)—an effect that was not observed in the stimulation “off” condition, suggesting that direct stimulation involves the middle temporal cortex during successful memory encoding.

Although the present study utilized stimulation parameters (i.e., frequency and pulse characteristics) similar to those used in previous studies (Suthana et al., 2012; Jacobs et al., 2016; Hansen et al., 2018), there are some discrepancies between the present and earlier work; these can probably be attributed to minor differences in stimulation parameters (i.e., increased duration and intensity) as well as different memory paradigms. While our study tested associative word-pair memory, an earlier study tested single-word item memory (Jacobs et al., 2016).

There is thus the possibility that task-related differences in hippocampal activity have affected the behavioral outcome of the hippocampal stimulation. A robust body of evidence indicates that the hippocampus supports the encoding of associative or relational information (Davachi, 2006; Diana et al., 2007; Mayes et al., 2007; Battaglia et al., 2011; Olsen et al., 2012; Yonelinas, 2013). Moreover, selective hippocampal lesions severely impaired associative memory rather than item memory itself in humans (Turriziani et al., 2004) as well as in primates (Zola-Morgan et al., 1986; Pascalis and Bachevalier, 1999). Differences in excitability of neurons could also play a key role in determining the behavioral outcome of stimulation effects. Indeed, neuronal activity in the human hippocampus is significantly higher for associative paired-item memory than for single-item memory (Cameron et al., 2001).

As mentioned earlier, previous studies using non-invasive stimulation have described the role of the hippocampus in associative memory (Wang et al., 2014; Wang and Voss, 2015) and memory precision (Nilakantan et al., 2017), and demonstrated its selective influence on associative vs. item memory (Tambini et al., 2018). However, these studies did not provide confirmatory evidence of hippocampal-dependent functions, since non-invasive stimulation is limited to delivering stimulation specifically to the hippocampus itself. Our current findings, however, provide direct evidence for a causal role of the human hippocampus in associative memory. Further expanding the knowledge provided by prior studies, our results suggest that direct hippocampal stimulation can enhance hippocampal-dependent associative binding.

Stimulation intensity and duration may be important regarding whether memory will be disrupted or enhanced by the activity in the hippocampus. A previous study suggested that optimal stimulation is based upon the current level, rather than the frequency of stimulation (Hescham et al., 2013). Furthermore, the duration of stimulation may lead to changes in the total energy delivered to the tissues (Moro et al., 2002). Additionally, different stimulation parameters have been associated with both increases and decreases in downstream brain activity in previous electrophysiology and functional MRI (fMRI) studies (Logothetis et al., 2010). We speculate that the positive effect of stimulation on behavior in the present study may be due to both the memory task and the precision of the stimulation parameters. As we were unable to directly address the controversy presented in the literature, further studies are required to explore the precise effect of stimulation on neural activity and variations in behavioral outcomes.

Our behavioral results indicate that hippocampal stimulation enhances memory performance, particularly recollection ability that retains specificity, and that the level of detail depends on the hippocampus. The hippocampus integrates elements of memory representations associated with the qualitative aspects of an event during encoding (Yonelinas, 2013), and it is known to be crucial for recollection, but not for familiarity (Davachi and Wagner, 2002; Giovanello et al., 2003; Addis et al., 2004). Previous neurophysiological and neuroimaging studies have indicated that the hippocampus is an essential region for remembering context-specific details (recollection; Eldridge et al., 2000; Moscovitch and McAndrews, 2002). One non-invasive stimulation study reported that hippocampus-targeted cortical stimulation enhances highly specific memory recollection (Nilakantan et al., 2017). Taken together, these findings demonstrate that direct stimulation of the hippocampus during encoding boosts memory recollection.

Furthermore, our results reveal that hippocampal-dependent memory is correlated with the theta activity of the lateral temporal cortex during memory encoding in the stimulation “on” condition. A previous functional MRI (fMRI) study investigating associative memory tasks indicated that the hippocampus supports strong recollection, with additional contributions from several neocortices (Wais, 2011). There is growing consensus that direct electrical stimulation regulates the physiology across a network connected to the stimulation site (McIntyre and Hahn, 2010; Kim et al., 2016; Ezzyat et al., 2017). Stimulation may thus be mediated via cortico-hippocampal communication (Acheson and Hagoort, 2013; Wang et al., 2014).

The present study focused on activity changes in the low-frequency band (i.e., theta rhythm), for several reasons. For example, low-frequency power may maximize excitability and enhance subsequent memory encoding in cortical regions in the presence of word pairs (Lakatos et al., 2008; Haque et al., 2015). Theta rhythms generate large and synchronous membrane-potential fluctuations in many neurons throughout brain-wide networks (He et al., 2008; Buzsáki et al., 2013). Hence, the rhythm may be propagated to distant brain regions via long-range communication (Gloveli et al., 2005). Accordingly, our data revealed a concomitant increase in theta power in the temporal cortex during correctly remembered trials in stimulation “on” conditions. In particular, the hippocampal stimulation enhanced recollection ability. Although recollection was not specifically tested as in the present study, a previous study pointed out orchestration in theta phase-synchrony between the MTL and a distributed neocortical memory network for vividly remembered experiences (Fuentemilla et al., 2014).

Despite their critical role in human cognition, distributed networks of oscillatory activity in memory have remained largely uncharacterized. Our findings reveal stimulation-mediated theta oscillatory changes in the human temporal neocortex, thereby broadening our understanding of stimulation-induced neural correlates in one of the distributed networks that may act together with the hippocampus.

The present study has some limitations that should be noted, including the limited range of targets and parameters explored. Furthermore, as we only investigated six patients, we cannot claim with any certainty that the statistical power was sufficient. Despite these limitations, however, we observed a consistent positive effect of stimulation in all six patients, using a verbal associative memory paradigm. Further studies involving larger samples of patients are required and more in-depth analyses considering variable stimulation parameters are warranted.

## Author Contributions

SJ, JK, and CC contributed to the study design and wrote the article. SJ and CC performed the study. SJ and JK analyzed the data. JK and CC obtained funding.

## Conflict of Interest Statement

The authors declare that the research was conducted in the absence of any commercial or financial relationships that could be construed as a potential conflict of interest.
